# Developing a clinical-pathological framework of long COVID-related fatigue applied to public safety workers

**DOI:** 10.3389/fmed.2024.1387499

**Published:** 2024-07-17

**Authors:** Adriana Lofrano-Porto, Susanne D’Isabel, Denise L. Smith

**Affiliations:** ^1^Molecular Pharmacology Laboratory, Health Sciences School, University of Brasilia, Brasilia, Brazil; ^2^Endocrine Diseases Clinics, University Hospital of Brasilia, Brasilia, Brazil; ^3^First Responder Health and Safety Laboratory, Department of Health and Human Physiological Sciences, Skidmore College, Saratoga Springs, NY, United States

**Keywords:** fatigue, COVID-19, long COVID, PASC, post-COVID conditions, ME/CFS, public health, public safety

## Abstract

In the wake of the COVID-19 pandemic, millions worldwide are still struggling with persistent or recurring symptoms known as long COVID. Fatigue is one of the most prevalent symptoms associated with long COVID, and for many it can be debilitating. Understanding the potential pathological processes that link fatigue to long COVID is critical to better guide treatment. Challenges with diagnosis and treatment are reviewed, recognizing that post-COVID fatigue does not always present with corroborating clinical evidence, a situation that is frustrating for both patients and healthcare providers. Firefighters are a group of public safety workers who are particularly impacted by long COVID-related fatigue. Firefighters must be able to engage in strenuous physical activity and deal with demanding psychological situations, both of which may be difficult for those suffering from fatigue. Disruption in public safety worker health can potentially impact community welfare. This review creates a framework to explain the clinical-pathological features of fatigue resulting from long COVID, addresses diagnosis and treatment challenges, and explores the unique impact fatigue may pose for public safety workers and their organizations.

## 1 Introduction

COVID-19 is a respiratory illness caused by infection with SARS-CoV-2, the newest of seven coronaviruses known to infect humans. The respiratory tract is believed to be the main site of virus replication, particularly the lungs. For most, acute infection results in asymptomatic or mild-to-moderate illness characterized by a range of influenza-like symptoms such as fever, chills, cough, shortness of breath, muscle and headaches, fatigue, among others. In some cases, however, acute COVID-19 causes more serious illness that can require hospitalization. In the most severe instances, infection results in systemic disease that leads to multiorgan failure and death (1). And, for a substantial number of individuals, acute illness does not resolve completely and results in a condition known as long COVID.

COVID-19 led to millions of deaths and has disrupted the lives of hundreds of millions of people globally. Although the World Health Organization (WHO) declared an end to the COVID-19 public health emergency, the threat from COVID-19 is not over (2). Long COVID and, in particular, the nearly ubiquitous symptom of fatigue that accompanies the condition, remains a complex challenge about which much still needs to be understood in order for clinicians to help alleviate suffering.

The seemingly mysterious condition that was plaguing many individuals in the months following the onset of the pandemic was first identified as “long COVID” by patients who were struggling with unresolved illness and created an online network to share stories and seek advice. Long COVID, which has been variously termed Post-Acute Sequelae of SARS-CoV-2 infection (PASC) and Post-COVID Conditions (PCC), is currently defined by WHO as persistent or new symptoms 3 months after infection with SARS-CoV-2 that continue for at least 2 months in the absence of other explanations (3). Symptoms can also remit and then recur (3, 4). Since its emergence, defining this novel and multifactorial medical condition has challenged scientists, health care providers, and patients alike. As knowledge continues to evolve, it is likely that the terms and definitions will continue to evolve and, hopefully, so too will treatment options.

Although an exact global frequency for long COVID remains undetermined, it is estimated that 10–20% of people infected with SARS-CoV-2 develop this condition (3). In the latest wave of the Centers for Disease Control and Prevention’s Household Pulse Survey (April–May 2024), among U.S. respondents who had ever had COVID-19, 30.6% reported experiencing long COVID and 10.1% were still experiencing long COVID (5). In the European region, approximately 36 million people (or 1 in 30) had symptoms associated with long COVID in the first 3 years of the pandemic (6). Overall, the incidence of long COVID is estimated to be 10–12% among vaccinated cases, 10–30% of non-hospitalized cases, and 50–70% of hospitalized cases (7). Despite a higher prevalence in some of these groups, long COVID affects a wide range of people including those of any age, sex, ethnicity, socioeconomic status, occupation, and health status. It presents with different symptoms, affects multiple organs/organ systems, and displays a wide range of severity. The symptoms are highly heterogeneous, as reported worldwide in multiple studies, with fatigue identified as one of the most prevalent, occurring in about 85% of those with long COVID (8). Other commonly reported symptoms include: headache; shortness of breath; muscle and joint pain and/or weakness; and cognitive impairment related-symptoms, such as “brain fog” and attention deficits (9, 10).

Other long COVID reviews have focused on major organ systems (e.g., cardiovascular, respiratory, neural, immune) (11–17), or specific disease states (e.g., heart disease, diabetes) (18–22), and although some reviews have focused on long COVID-related fatigue itself (23–25), none present a comprehensive framework that extends understanding to the impact of fatigue on public safety workers. Additional investigation of fatigue related to long COVID is imperative given its prevalence. In fact, a large international study analyzing clinical manifestations across 10 international cohorts found 31 unique clinical features with fatigue being the most common symptom (median 45.1%) (26).

Fatigue can be debilitating for those suffering from it, eclipsing mere tiredness, and imposing a crushing sense of exhaustion, in some cases making everyday activities impossible. It also does not manifest in the same manner for everyone: for some it is a persistent “heaviness,” for some it is cognitive sluggishness, for some it is a “crash” after any exertion. Rest does not cure this fatigue. Fatigue is difficult to diagnose and, unfortunately, treatment options are limited, which frustrates both patients and clinicians (27, 28). Although clinicians have encountered post-viral fatigue before with similar diagnostic and treatment challenges, the magnitude of the post-COVID crisis is particularly concerning given the sheer number of people affected, the strain on the health care system, and the potential impact on the workforce. In particular, it is rational to presume that public safety personnel (e.g., firefighters and police officers) who grapple with long COVID-related fatigue face a unique burden due to the high psychological and physical demands of their job. However, specific data among this population remain scarce. Nonetheless, the importance of considering the impact of long COVID-related fatigue for public safety workers is obvious given the strenuous nature of their work and the fact that impaired work performance may jeopardize public safety.

Thus, the purpose of this narrative review is to provide a comprehensive clinical-pathological framework that synthesizes the literature about long COVID-related fatigue by: reviewing pathophysiological causes and symptom clustering; discussing clinical dimensions and management of long COVID-related fatigue; and, considering this health challenge within the context of public health and, specifically, how it may impact public safety occupations.

## 2 Framework for long COVID-related fatigue and its impact on public safety workers

**Figure 1:**
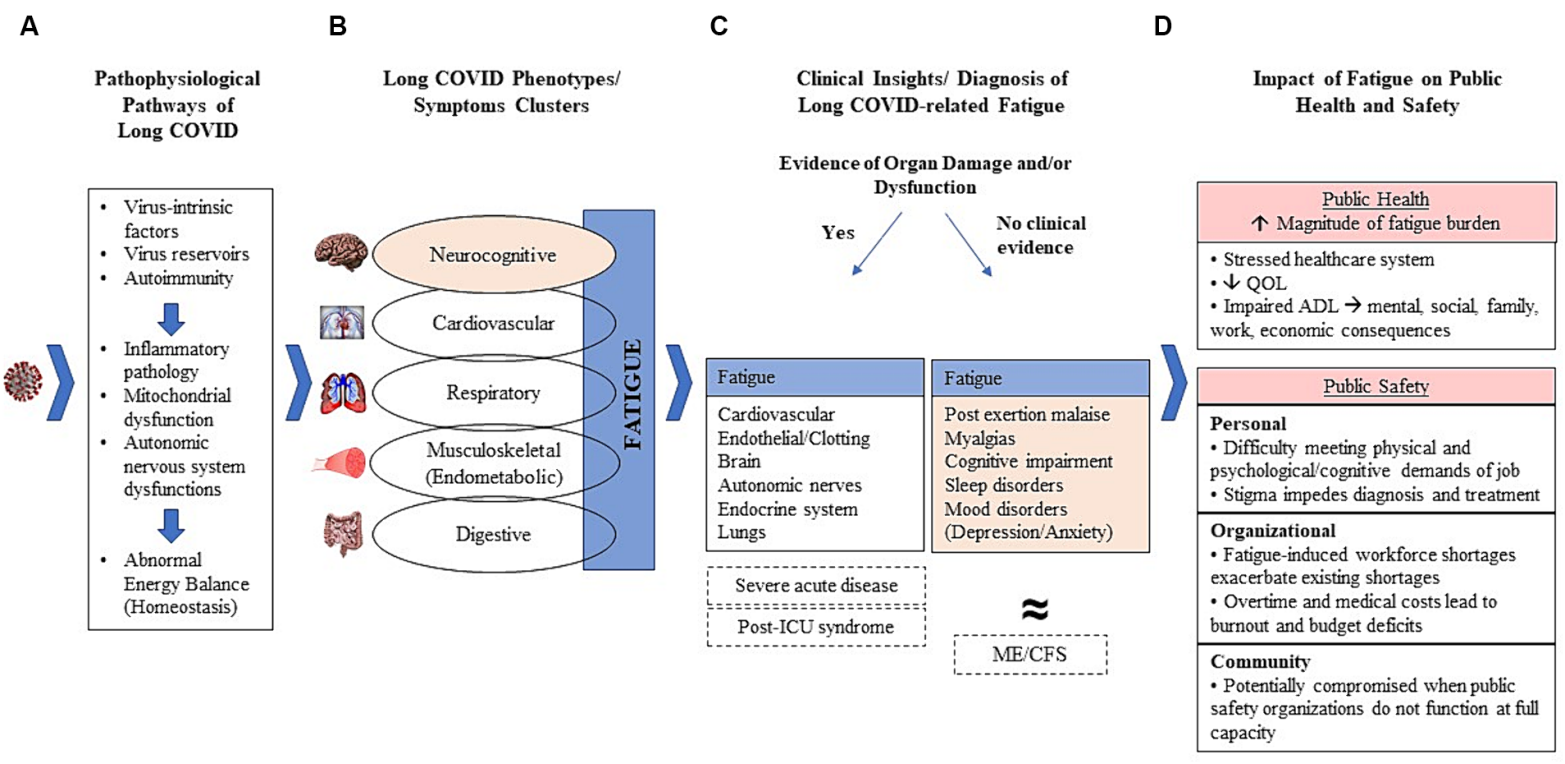
Clinical-pathological framework of long-COVID-related fatigue. **(A)** Potential pathological mechanisms leading to long COVID; **(B)** Major phenotypes and symptom clusters with fatigue central to all phenotypes and often existing as a cardinal manifestation itself; **(C)** Proposed initial diagnostic stratification of long COVID patients according to clinical evidence of organ damage and/or dysfunction; **(D)** Impact of fatigue on public health and safety.

Figure 1 presents a framework to systematically consider long COVID-related fatigue with a focus on public safety workers. This figure, and the sections that follow, highlight the varied pathophysiological consequences associated with long COVID (Figure 1A), the multiple long COVID phenotypes/clusters and the role of fatigue in these (Figure 1B), a clinical approach to those who present fatigue with and without evidence of organ damage (Figure 1C), and the potential impact on public safety workers (Figure 1D). Although relatively little clinical research has been done on public safety workers, it has been universally accepted that long COVID-related fatigue may linger for months and can substantially impair a person’s ability to work, irrespective of the type and demands of specific occupations. However, disability protection for workers is often lacking (29). The compilation of data that support the framework are presented in the following sections.

## 3 Major pathophysiological pathways of fatigue

SARS-CoV-2 can damage organs in diverse body systems, and a growing worldwide effort is ongoing to better characterize the specific pathophysiological pathways for persistent conditions associated with long COVID (7). However, mechanistic and immunopathologic studies have shown considerable heterogeneity across subjects, thus limiting generalized conclusions regarding disease mechanisms. Briefly, the best available evidence suggests that immunopathogenic derangements induced by the acute SARS-CoV-2 infection are key features of resultant pathophysiology (30). As depicted in Figure 1A, it has been proposed that viral intrinsic mechanisms circumvent the immunological defenses and act as triggers and/or drivers of systemic or organ-specific inflammatory pathology, disruptions in the autonomic nervous system, mitochondrial function, and impaired energy balance (30, 31).

Indeed, the existence of an active SARS-CoV-2 reservoir in the nervous system has been demonstrated through different experimental approaches (32). Resultant neuroinflammation and neuronal injury underscore the neuropathology of long COVID-related fatigue (33). Furthermore, viral-induced inflammasome activation promotes TGF-beta and type I interferons dysregulated signaling, among other immune-mediated processes that trigger the generation of autoantibodies and ultimately play key roles in acute disease severity and progression to long-COVID (34, 35).

In short, fatigue is a manifestation of a global homeostatic imbalance, which shows up in various post-COVID conditions (36). Any of these pathological changes, or a combination of them, may lead to multiple organ dysfunction.

## 4 Fatigue as a component of long COVID phenotypes

Fatigue is a particularly challenging feature of long COVID in terms of pathology as it is presumed to reflect a multisystem impairment itself. A considerable amount of research has employed different strategies to cluster symptoms experienced by patients into clinical phenotypes, with many strategies identifying neurocognitive, cardiovascular, respiratory, musculoskeletal, and digestive phenotypes (26, 30, 36–39). Importantly, fatigue is the most commonly reported symptom of long COVID and is evident across phenotypes (Figure 1B) (26, 36–42). The reasons for fatigue are complex and unclear and may be related to organ damage in some phenotypes (cardiovascular, respiratory, musculoskeletal, digestive/gut) (37, 38). Although fatigue is a symptom of each phenotype, some also consider it to be a unique phenotype or clinical entity in its own right (Figure 1B) (39, 42). There has been increasing awareness of the potential benefit of defining phenotypes of individuals presenting symptomatology consistent with long COVID in a way that enables a clinical stratification of patients (36–38). However, clinical stratification through clustering analyses has been accomplished through different research methods, leading to multiple categorizations and limiting comparability in clinical trials, which hampers clear guidance to healthcare providers (26, 37, 38).

## 5 Clinical characterization of long COVID-related fatigue

Technically, fatigue refers to “the subjective human experience of physical and mental weariness, sluggishness, low energy, and exhaustion. In the context of clinical medicine, fatigue is most typically and practically defined as difficulty initiating or maintaining voluntary mental or physical activity” (43). However, defining fatigue is a challenge as it is conceptually multidimensional. Fatigue comprises physical, mental, cognitive, emotional and motivational domains (44, 45). It involves multiple biological systems, although it most often presents under a neural-related dimension (46, 47).

### 5.1 Clinical presentation

Usually, fatigue is reported within the context of a specific medical condition, thus it can be accompanied by other general manifestations such as pain, sleep impairment, depression, and cognitive dysfunction. Fatigue is a central feature of the neurocognitive dysfunction associated with the long COVID phenotype, as well as being present in other phenotypes. Regardless of its pathophysiological basis, fatigue has a profound effect on an individual’s functioning at home or work and their ability to socialize (46). The negative impacts of fatigue encompass loss of productivity at work, occupational hazards, suicidal ideation and medication abuse which can be life threatening and may exacerbate neurocognitive impairment and psychiatric conditions like depression/anxiety (37, 38, 48, 49). Paradoxically, in general clinical settings, fatigue itself is frequently brought to medical attention only when the underlying cause is unclear, when it fails to remit, and/or when its severity is disproportional for the related trigger, if known (28). Since the COVID-19 pandemic, however, fatigue has been increasingly recognized as a serious, potentially disabling, multidimensional post-COVID condition.

The multiple dimensions of fatigue in different individuals further complicate its recognition and contribute to it being underreported and even undertreated. Often reported as a “feeling” by the patient, or an “unspecific symptom” by the health care provider, unrecognized and untreated post-COVID fatigue increases the proportion of newly disabled individuals thus resulting in labor shortages (50). Furthermore, like myalgic encephalomyelitis/chronic fatigue syndrome (ME/CFS), post-COVID fatigue may last for years, and it is presumed that a substantial number of individuals engaged in strenuous occupations may never return to work. Fatigue is a specific concern among public safety personnel due to the physically demanding nature of their work. In addition, among the many clinical manifestations that cluster with fatigue, cognitive dysfunction, specifically, can impair situational awareness and complex problem-solving skills that are necessary to safely function in a complex, life-threatening environment.

### 5.2 Diagnosis

The first step to assess any persistent health issues, including long COVID, in the clinical setting is to perform a comprehensive medical history and physical examination, with an accurate index of suspicion, being particularly attentive to:

- Characteristics of the acute COVID illness, including timeline, severity of symptoms and complications, treatments/medications needed;
- Current symptoms and severity, timeline after acute infection, medications in use, daily living activities, variable persistent health issues that may have arisen over time;
- COVID-19 testing and previous results of lab work-up and imaging;
- Comparison with previous pre-infection health status (51).

**Figure 2:**
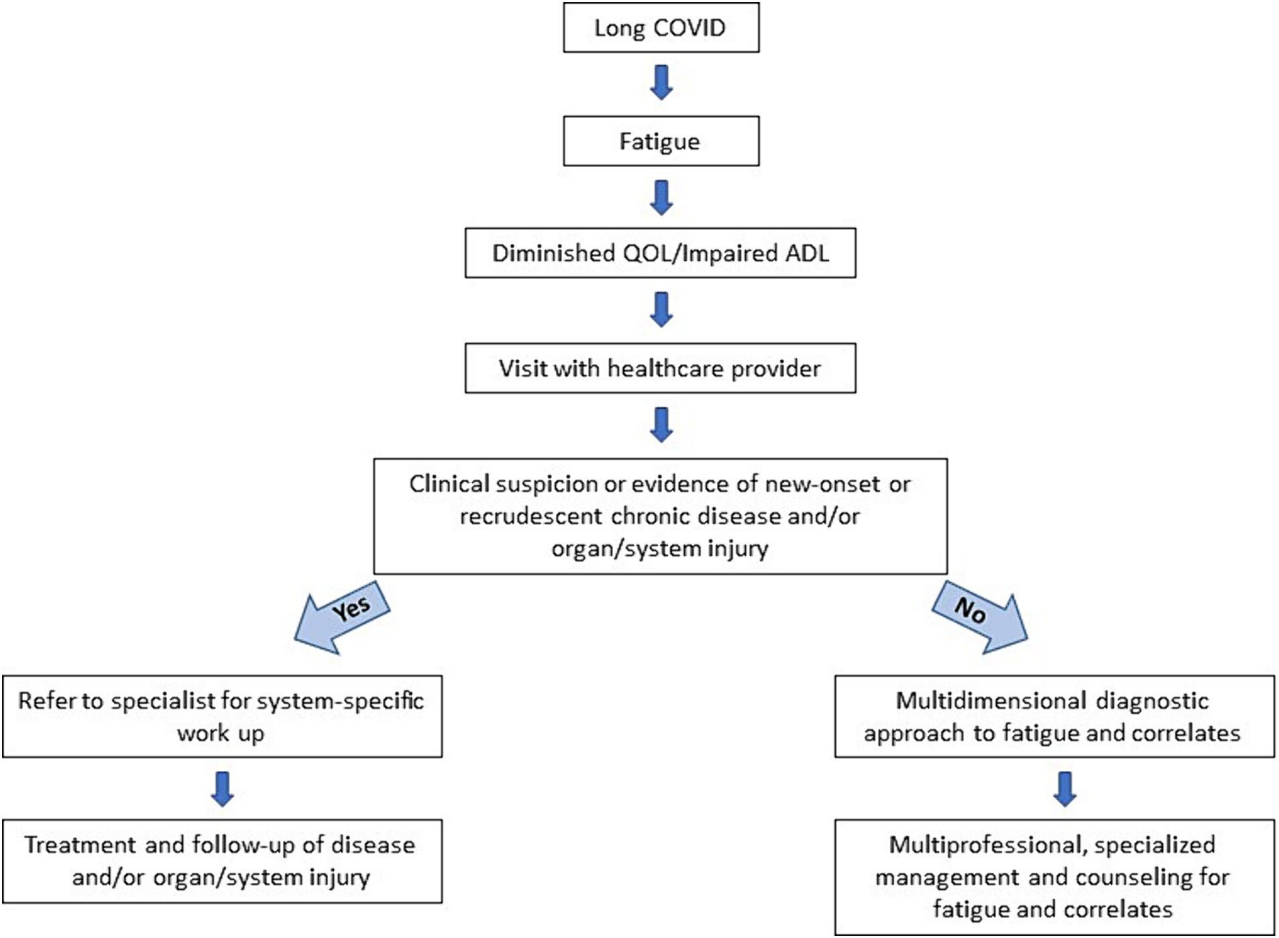
Proposed clinical flow sheet to guide, diagnose and treat long COVID-related fatigue.

Diagnosis may be facilitated by considering long COVID cases within the context of two major clinical scenarios (Figures 1C, 2) (51):

1. Individuals with persistent or new fatigue as a major manifestation, often accompanied by cognitive impairment, headaches, disrupted sleep, myalgia/arthralgia, post-exertion malaise, orthostatic intolerance, **but with no obvious clinical evidence of major organ injury** (for example, lung fibrosis, cardiomyopathy, stroke, kidney failure). These individuals have been recognized as part of the long COVID disease group in which fatigue and its correlates prevail.
2. Individuals with persistent or new fatigue and **clinical evidence of tissue/organ damage.** Importantly, some mild or subclinical organ dysfunction might not be obvious at the time of the first evaluation, but is suspected, so a closer follow-up is necessary given the greater potential to progress to long-term organ dysfunction. This group also includes individuals that have **new onset, recrudescent or aggravation of chronic diseases,** such as diabetes mellitus, cardiovascular diseases, stroke, or respiratory failure. These conditions often cause fatigue and are associated with a substantially increased risk of death in the first year following acute infection, compared to matched persons with no evidence of COVID-19 (52).

In addition to these two categories, there is a smaller number of individuals who have had severe acute disease and continue to manifest fatigue and other symptoms similar to what occurs in other serious post-critical illness with the need for lifelong rehabilitation and medical care (post-intensive care unit syndrome-PICS) (52).

The lack of precise diagnostic biomarkers complicates the clinical approach to the individual with long COVID, particularly in those who have unexplained persistent fatigue as the only or predominant symptom. In spite of that, evidence-based, step-by-step diagnostic approaches, starting from a thorough and welcoming clinical consultation, supplemented by routine lab-work (complete blood count, electrolytes, urea/creatinine, and liver function tests) efficiently map the general health status of the patient. In patients with signs of more severe disease, complications or suspicion of organ damage, additional tests to evaluate signs of organ damage are necessary (51, 53).

Data from COVID-19 specialized clinics in the U.S. support a deeper evaluation of patients with clinical suspicion of long COVID, especially in those with a neurocognitive phenotype and with fatigue as a major symptom (51). The diagnostic approach in these cases is enriched using standardized screening measures to better characterize physical and mental impairment. A series of tests recommended by the Critical Care International Consensus Conference are useful to predict and identify physical and mental impairments, including fatigue and related symptoms. In brief, testing consists of a combination of screening tools to detect long-term cognition, mental, and physical function (54). Proposed tools include the Montreal Cognitive Assessment (MoCA), 6-min walk and EuroOol-5E-SL which include assessments of mobility, self-care, usual activities, and anxiety/depression (55). In addition, use of the Patient-Reported Outcomes Measurement Information System (PROMIS^®^) offers a standardized scale to collect information about long COVID that would facilitate comparisons of data (56). Research addressing the correlation of neuroimaging findings and post-COVID fatigue have improved our understanding of its neuropathology, however as results remain inconclusive, neuroimaging studies are not indicated for screening or diagnostic purposes of fatigue in clinical scenarios (57–59).

Additionally, a series of recommendations to assess post-COVID fatigue have been proposed to address the severity of perturbation of daily living activities. A detailed inquiry about factors that exacerbate fatigue is essential for the identification of underlying conditions that may be masked, such as sleep and mood disorders, and mild, undiagnosed cardiopulmonary, autoimmune and endocrine disorders. Through systematic and individualized protocols for clinical investigation of fatigue, functional status of long COVID sufferers can be determined, while these conditions can be excluded or closely monitored (60).

#### 5.2.1 Similarities between long COVID-related fatigue and ME/CFS

Researchers worldwide have noted clinical-pathological similarities between long COVID and ME/CFS, a chronic and debilitating predominantly neuroimmune condition that is often lifelong (27, 28, 52). Indeed, recent studies have found that a substantial proportion (13–58%) of patients with long COVID also meet the diagnostic criteria for ME/CFS (27). Interestingly, in self-report surveys, both over-diagnosis and under-diagnosis were evidenced (52). Thus, recent initiatives to develop robust, validated instruments to measure, or at least estimate, the burden of functional impairments that associate with fatigue in long COVID and/or ME/CFS patients are badly needed.

ME/CFS has been correlated with previous viral infections such as Epstein–Barr virus, SARS-CoV-1, H1N1 Influenza, Cytomegalovirus, Herpes-6, and others (47). In general, post-COVID conditions share clinical-pathological characteristics of this challenging chronic illness. Like long COVID, diagnosis of ME/CFS is often not straightforward and its pathophysiological basis remains uncertain and variable. Several potential disease mechanisms have been proposed for ME/CFS that mirror long COVID-related fatigue, including neuroinflammation, immune derangements, mitochondrial dysfunction, endothelial and muscle disturbances, hypoperfusion, abnormalities of the peripheral nervous system, and a broad homeostatic imbalance (7, 28, 47, 58, 61). Although there is increasing evidence that many of these mechanisms are common in long COVID and ME/CFS, it remains unclear whether and how these disease pathways may be related and orchestrated.

### 5.3 Therapeutic approach

The American Academy of Physical Medicine and Rehabilitation has developed a multidisciplinary collaborative consensus guidance statement on the assessment and management of fatigue following COVID-19 illness (55). It has served as a useful reference for COVID-19 specialized clinics to guide post-COVID-related fatigue protocols. Unfortunately, however, there continues to be a lack of resources specific to the challenges faced by public safety workers.

Therapeutic strategies to manage physical activities, hobbies, diet, social and familial interactions are encouraged. It is also important to be attentive to emotional factors. Currently, a general guidance for management of patients diagnosed with persistent fatigue and impaired functional status starts with educational strategies for conserving energy. Encouraging good sleep hygiene for restoring rest is foundational. Beyond that, one method known as the “four-P” approach (Planning, Pacing, Prioritizing and Positioning) focuses initially on daily living activities. Importantly, Pacing is a learned skill that helps patients obtain enough energy to complete activities. For example, an individual suffering from fatigue will recover faster if he/she works on a task until tired rather than exhausted. In other words, going for “the big push” will probably result in longer recovery. This aspect of the “four-P” approach is important for workers diagnosed with fatigue. Tips for pacing include to break activities into smaller tasks spread throughout the day, adjust different parts of an activity to reduce the energy demands, and take rests between activities (recharge) when possible. It is important to note that following this guidance may be difficult at work, especially when work requires strenuous effort, or when public safety is at risk.

Individualized advice/education programs are based on the level, daily pattern, and dimensions of fatigue, given symptoms may fluctuate over days and as levels of activities increase. Symptoms should be continuously assessed and compared with pre-illness functional status (e.g., decline in exercise tolerance, weakness, or reduced mobility) (55, 62). Some patients are unable to tolerate progression and experience worsening and/or post-exertion malaise. When individuals are unable to make progress, referral to outpatient rehabilitation specialists may be needed. Individuals with poor exercise performance compared to pre-acute infection status should be re-evaluated for mild, undiagnosed, or evolving cardiopulmonary, endocrine-metabolic and/or neurocognitive pathology. Monitoring cardiorespiratory fitness may be especially important in public safety groups with minimum fitness for duty standards (63). Referral to specialized cardiopulmonary rehabilitation programs may be necessary. Standardized functional assessment tools can also be used to monitor the patient’s progress over time. A functional assessment tool specific to COVID-19 has been developed using an ordinal scale to capture a range of limitations from none to severe (60). Although not yet validated, the use of a scale allows a more robust assessment of the impact of illness beyond binary measures such as mortality and also provides the opportunity to document change (60). Importantly, graded exercise therapy has been only cautiously proposed for fatigue related to long COVID as reports of aggravation of fatigue, mostly post-exertional malaise, have been reported (64). Because of the risk of post-exertional malaise and muscle damage, it is recommended that physical activity should be closely supervised (31).

A standard healthy diet and adequate hydration is recommended for all patients and may be adapted in individual cases. So far, there is neither enough evidence to support the benefits of a fatigue-specific diet plan, nor the use of nutritional supplements. It is likely that people engaged in strenuous activities who experienced loss of muscle mass and/or severe muscle weakness would benefit from individualized nutritional support and specific rehabilitation exercises (55, 65). Despite the lack of research to support a therapeutic role for a specific diet intervention in addressing long COVID-related fatigue or ME/CFS, there has been increasing evidence of the health benefits of plant-based diets, mostly the Mediterranean diet, as a therapeutic adjuvant in a variety of inflammatory diseases, such as multiple sclerosis, systemic autoimmune diseases, and obesity (66, 67). Given the prevailing hypothesis that long COVID develops from an increased systemic inflammatory response, it is important to continue research initiatives that examine the effects of a Mediterranean diet and other non-pharmacological strategies, such as considering the inflammatory index of nutrients and promotion of favorable microbiome signatures (68–70).

In spite of increasing efforts to discover effective pharmacological treatments for long COVID, definitive evidence for the use of pharmacological agents is still lacking. Several clinical trials are underway, including some that were originally designed for ME/CFS. Many new medications target specific pathological pathways associated with long COVID. However, given the multiple pathological pathways, it is likely that pharmacological treatment will need to vary for patients presenting with various phenotypes. Determining which drug will work for which patient or which phenotype/disease presentation has been challenging.

The most promising medications that may be useful for the treatment of patients with long COVID include:

- **SARS-CoV-2 monoclonal antibodies**. These proteins target viral circulating spike proteins that sit in viral reservoirs and induce the immune system to react as if it is still fighting acute COVID-19. Preliminary results with a small series of patients have recently shown a striking rapid and complete remission of the symptoms (71), and larger scale trials are ongoing.
- **Nirmatrelvir plus ritonavir.** These oral antivirals block a key enzyme for virus replication. Partial results showed opposite responses, ranging from life-changing for some patients to ineffective for others, while some moderate side effects have been reported (72).
- **Metformin**. This globally available, low cost, and safe anti-diabetic reduced long COVID incidence by 41% when taken on an outpatient basis during the acute infection, compared to placebo (73). Its immune-modulatory effects have been significantly useful to control systemic inflammation that contribute to long COVID.
- **Selective Serotonin Reuptake Inhibitors (SSRIs)**. This worldwide popular class of antidepressant molecules has recently arisen as a potential treatment to restore serotonin levels in long COVID sufferers, once compelling evidence of an association between viral inflammation-driven serotonin depletion and long COVID have been unraveled (74).
- **Low-dose Naltrexone (LDN).** This oral μ-opioid receptor antagonist has been previously shown to have immune-modulating properties. In a cohort of 59 patients with long COVID, the use of LDN in individualized doses (titration ranging from 0.5 to 6.0 mg/day) was associated with improvements of fatigue and functional status without serious adverse effects (75). In another pilot study using LDN (4.5 mg/day) plus supplementation with NAD+, the authors found improvements of fatigue and a significant increase from baseline in SF-36 survey scores after 12 weeks of treatment. Randomized clinical trials are needed (76).
- **Aripiprazole.** This Dopamine D2 receptor agonist has previously demonstrated the ability to modulate neuroinflammation, microglia activation and cell death in animal models and humans. In a retrospective cohort from a specialized clinic, medical records from 101 individuals who met the diagnosis of ME/CFS received off-label, low-dose aripiprazole (0.2–2.0 mg/day). 74% of the subjects experienced improvement of fatigue, brain fog, post-exertional malaise and other related symptoms. Randomized clinical trials are needed (77). Recently, a protective effect of Aripiprazole against fatal outcomes in individuals with severe acute infection has been suggested, possibly associated with its effects on immunological and inflammatory pathways (78).

The ability to return to work should also be individually based and will likely vary depending upon baseline pre-illness functional status, the severity of fatigue, occurrence of complications, and the intensity of work activity. For most patients, gradual resumption of exercise/work as tolerated is recommended, starting at a low-intensity level and slowly increasing activity over several weeks. Workers engaged in strenuous activities who eventually have new or progressive symptoms during resumption of work activities, or difficulty advancing to pre-COVID-19 activity levels, should have a follow-up clinical consultation and be considered for re-evaluation referral for cardiopulmonary testing (51, 60).

## 6 Implications for public health and safety

Long COVID-related fatigue poses a considerable threat to public health (Figure 1D). Over 18% of all adults in the U.S. have experienced long COVID, which means that millions of people continue to struggle with the effects of COVID-19 (5). Given that fatigue is the most cited symptom of long COVID, it is not an exaggeration to label post-COVID-19 fatigue an epidemic, as the magnitude far exceeds any previous experience we have had with other post-viral fatigue such as ME/CFS (37, 40, 41, 75). This will incur a substantial, and as yet underestimated, economic cost, as evidenced by the economic burden of neuropsychological conditions which demand similarly complex diagnostics, as well as complicated and long-term treatment (79). Therefore, a healthcare system that, worldwide, is taxed from COVID-19-related pressures that include provider shortages, supply shortages, patients with compounding needs, etc., must brace for future strain posed by a complicated and increasingly prevalent condition.

While the healthcare system appears vulnerable at this point, individuals suffering from long-COVID-related fatigue also feel vulnerable—even fragile. Quality of life can be sorely diminished for those suffering from fatigue, impacting the ability to perform even simple activities of daily living such as going to the grocery store or taking a shower. This has personal and population-based ripple effects, taxing social and family relationships, mental health, and ability to work. For some, different aspects of employment might become challenging; for others, work might be the only activity possible, causing home and social life to suffer; and, yet for others, work becomes impossible. This places an enormous stress on household finances and, unsurprisingly, further exacerbates family tensions and strains mental health.

Even prior to the COVID-19 pandemic there was a noted association between work stress and fatigue. Individuals who experience high levels of stress at work are more likely to seek care for mental health problems, sleep disturbances, and fatigue, compared to those who do not report high work-related stress (80). Indeed, the long-term health impacts of work-related stress, particularly among workers in strenuous occupations, are still underestimated. This condition may be even more concerning for workers that perform night-shift work who have increased risks for stress-induced physical and mental health problems (81, 82).

Work-related stress places increased demands on the human body, including an abnormally activated sympathetic nervous system and altered functioning of the hypothalamic-pituitary-adrenal axis, leading to abnormal cortisol secretion and consequent disrupted circadian rhythms (83). Indeed, chronic stress has been increasingly considered an additive risk factor for many negative health outcomes, such as cardiovascular disease, obesity and related dysmetabolic disorders (84, 85). Chronic stress is also associated with a variety of symptoms including fatigue, which may persist for years in affected individuals (86, 87). Furthermore, chronic stress exposure potentiates inflammatory processes and associated neuronal atrophy, mimicking, in many aspects, underlying long COVID pathology (88).

Therefore, in a real-world scenario where public safety workers are exposed to high levels of work stress that may combine with long-COVID-related fatigue, initiatives to improve healthcare for this population are needed. And, although each occupational cohort should be attentive to the enormous challenges their workers suffering from long COVID-related fatigue face, as an occupation that includes millions of employees worldwide, and one for which fatigue can have both personal *and* public safety implications, it is important to explore long COVID-related fatigue within the context of public safety personnel (Figure 1D). A physically strenuous and psychologically taxing occupation, workers in this field are especially vulnerable to the impacts of fatigue. Those struggling with fatigue may find physical exertion nearly impossible, or respond to calls and then experience post-exertion crashes. Beyond the physical demands of the job, many public safety personnel deal with psychologically harrowing situations. Mental demands of this nature and complex emergency scenes require a high level of cognitive functioning that might be compromised for those dealing with fatigue, especially those with neurocognitive phenotype. Ideally, those experiencing fatigue would see a healthcare provider for help. In the public safety field, however, there is a tendency to celebrate strength and stigmatize weakness; therefore, public safety personnel coping with fatigue may mask their problems from both coworkers and clinicians who determine their fitness for duty. And, even in the absence of stigma, if a public safety worker seeks help, fatigue is tricky to diagnose and treat, as described in this review.

From a public safety standpoint, the situation is also problematic. A full contingent of firefighters, police officers, emergency medical technicians, and other first responders is necessary to appropriately ensure the public’s safety. However, public safety organizations are still reeling from workforce shortages that have occurred since the pandemic. For example, there has been a loss of over 4,000 sworn officers across 182 U.S. law enforcement agencies since January 2020 and the U.S. Fire Administration cites recruitment and retention as primary challenges (89, 90). When positions go unfilled, communities are left vulnerable and public safety organizations will need to face the reality that post-COVID fatigue may aggravate this problem as struggling workers curtail their duties, delay a return to work, or remove themselves from the workforce entirely. Options to avoid this, however, are limited and introduce other problems. In order to meet community needs, public safety employers may require mandatory overtime to fill in for workers who are out with illness (backfilling) which can further burden existing workers and, ironically, compound the original problem by causing additional work-related stress and burnout (91). In addition to burdening workers, the budget implications of paying overtime to employees covering shifts and covering medical costs of workers creates an unsustainable financial position for the organization. And, this challenge must also be considered in the context of existing difficulties with recruitment and retention of public safety workers.

## 7 Conclusion

Long COVID-related fatigue is a growing and challenging health condition, at both an individual and a population level. A variety of issues coalesce to make post-COVID fatigue a particularly complex health problem to address. First, the condition presents a diagnostic conundrum as there are often no obvious signs of organ damage to suggest a cause and help with diagnosis. Second, although modern medicine has come to depend heavily on algorithms for treatment, fatigue presents with no biomarkers, thwarting this approach. Fatigue diagnosis is often one of exclusion versus inclusion of an underlying illness. It is based heavily on a combination of subjective and multidimensional clinical criteria. Third, the challenges of diagnosis can result in patients feeling stigmatized by clinicians, colleagues, and family members who, without definitive biological evidence, attribute the condition to psychological causes that can be dismissive. Fourth, even after the difficult road to diagnosis, treatment options are sparse and multi-disciplinary clinics for management of long COVID-related symptoms remain scarce.

Long COVID-related fatigue shares many similarities with ME/CFS, including treatment challenges. While this provides a potential opportunity to learn from a similar condition, research into ME/CFS remains largely underfunded, despite over a million people in the U.S. alone suffering from it (92). Post-COVID fatigue certainly affects more people than ME/CFS. Thus, in addition to the vast implications on personal health, post-COVID fatigue will strain the health care system, as well as the ability to fill critical public safety roles; thus, it is essential that research and clinical guidelines be advanced. Future research that explores the impact of long COVID-related fatigue specifically on public safety personnel, looking at the longer-term impact, is essential to provide a more robust understanding and fill gaps in current knowledge. While all workers may struggle with post-COVID fatigue, these challenges present unique concerns for workers in the public safety sector and for public safety at large.

Through this review, we developed a concise framework that compiles the clinical-pathological basis of fatigue in the context of long COVID sufferers, their clinical presentation and diagnosis, the current basis for therapeutic management, and insightful evidence-based information to call attention to the specificity of post-COVID-related fatigue in public safety personnel, which remains under investigated.

## Author contributions

AL-P: Writing – original draft, Writing – review & editing. SD’I: Writing – original draft, Writing – review & editing, Project administration. DS: Conceptualization, Writing – original draft, Writing – review & editing, Resources, Supervision.
